# Global Trends and Hot-Spots in Research on Virtual Simulation in Nursing: A Bibliometric Analysis From 1999 to 2021

**DOI:** 10.3389/fpubh.2022.890773

**Published:** 2022-04-25

**Authors:** Qian Zhang, Jia Chen, Jing Liu

**Affiliations:** ^1^Department of Neurosurgery, Xiangya Hospital, Central South University, Changsha, China; ^2^Xiangya Nursing School, Central South University, Changsha, China

**Keywords:** virtual simulation, nursing, bibliometric analysis, hot-spots, Citespace, VOSviewer

## Abstract

**Background:**

Virtual simulation has been widely used in nursing education and nursing training. This study aims to characterize the publications in terms of countries, institutions, journals, authors, collaboration relationships, and analyze the trends of virtual simulation in nursing research.

**Methods:**

Publications regarding virtual simulation in nursing were retrieved from Web of Science core collection. Microsoft Excel 2010, VOSviewer were used to characterize the contributions of the authors, journals, institutions, and countries. The trends, hot-spots and knowledge network were analyzed by Citespace and VOSviewer.

**Results:**

We identified 677 papers between 1999 and 2021. The number of publications grew slowly until 2019, after that, it got a sharp increase in 2020 and 2021. The USA, Canada and Australia were three key contributors to this field. Centennial College and University of San Paulo, University of Ottawa and Ryerson University were top major institutions with a larger number of publications. Verkuyl M was the most productive and highest cited author. Clinical Simulation in Nursing, Nurse Education Today, Journal of Nursing Education were the three productive journals. The foundational themes of virtual simulation research in nursing are “virtual learning during COVID-19, clinical nursing care, education in nurse practitioners, education technology”.

**Conclusion:**

Virtual simulation in nursing field has attracted considerable attention during COVID-19 pandemic. The research hotspot is gradually shifting from clinical nursing care to studies of nursing education using different virtual simulation technologies

## Introduction

Virtual simulation (VS) is defined by a kind of computer simulation systems involving real people operating simulated systems, it could provide immersive, highly visual, 3D characteristics that allow the users to explore the role and changes within a seemingly real or physical world (1). Generally, it included three technologies [e.g., virtual reality (VR), augmented reality (AR), and mixed reality (MR)] (2, 3). In the last decades, with the adoption, application, and maturity of these technologies, nursing educators have increasingly used VS for nursing education and clinical training. For example, Girao et al. (4) developed a serious virtual reality game for medication preparation and administration training. Weston and Zauche (5) conducted a virtual simulation with clinical practice for pediatric nursing students. Chao et al. (6) applied the immersive three-dimensional interactive video program to help nursing students better acquire nasogastric tube feeding skills. Compared to traditional teaching approaches, VS has the advantage of minimal requirement of medical equipments or consumables. What's more, the sense of immersion is enhanced by wearable displays, interactivity, and haptic devices. And users can practice it anywhere and anytime as long as the device is available (1). In 2018, Tiffany and Forneris (7) forecast that the adoption of VR in nursing education will from the current 10% use to 45% in the next 5 years. Specifically, The COVID-19 (coronavirus disease) pandemic has spurred a transition from face-to-face teaching to virtual learning (5, 8). As a burgeoning area, more and more educators and policymakers prefer using VS to assist teaching. To this end, there is an urgent need to investigate the performance (e.g., primary contributors and highly cited articles) and scientific map (collaborations, research themes, and trends) to show the past, present, and future direction in this field.

Bibliometric analysis is widely accepted for reviewing big data of articles in a field or publications in a journal using quantitative techniques (9, 10). It usually applies bibliometric tools (e.g., Bibliometrix R, Gephi, Pajek, CiteSpace, Leximancer, and VOSviewer) to analyze the publication trend, the top articles, primary contributors, and major theme and frontier topics in a given field (11–13). There are several existing bibliometric analysis about nursing topics (14–16). However, no study focused on bibliometric analysis to provide a state-of-the-art review for VS research in nursing field. Therefore, in this study, we utilized the popular bibliometric tools, Citespace and Vosviewer (17, 18), to comprehensively analyze VS research in nursing based on the Web of Science Core Collection (WoSCC). We hope this paper will uncover the following research questions (RQ).

**RQ1**. What is the publication trend for virtual simulation research in nursing?

**RQ2**. Which are the most influential articles and primary contributing authors, institutions, countries, journals for virtual simulation research in nursing?

**RQ3**. What are the potential collaborators (author, institutions, countries/regions) for virtual simulation research in nursing?

**RQ4**. What are the major themes and frontier topics for virtual simulation research in nursing?

## Materials and Methods

### Aims

The aims of this study are as following: (1) to uncover the major contributors (e.g., countries, institutions, journals, authors, articles) in VS related to nursing research. (2) To analyze the co-operation relationships in this field. (3) To map the knowledge network and identify the frontier topics, and point the future directions in this field.

### Design

A descriptive bibliometric analysis of publications in virtual simulation related to nursing research.

### Sample/Participants

The data in this research were retrieved from Web of Science database, so no participants were involved.

### Search Strategy

The advanced search was performed using Web of Science (Thomson Reuters, New York, USA) on March 15th, 2022. Formula: (((TS= (“virtual simulation” or “virtual realit^*^” or “virtual reality simulation” or “virtual learning” or “augmented realit^*^” or “mixed reality”) and TS= (“nurs^*^”)) AND DOP=(1999-01-01/2021-12-31)) AND DT=(Article OR Review)) AND LA=(English) was used to screen out publications associated with VS in nursing. Two team members (Qian Zhang and Jing Liu) searched and screened the database independently. Any discrepancies were resolved by discussion with Jia Chen until consensus was reached.

### Inclusion Criteria

(1) Peer-reviewed articles involving VS related to nursing
(2) Original articles and review articles
(3) Written in English.
(4) Web of Science core collection (WoSCC).

### Exclusion Criteria

(1) Unpublished papers
(2) Articles required a manual research.

### Data Extraction and Analysis

The following bibliometric parameters were extracted, such as title, keywords, journal, publication year, citation counts, citations per paper, H-index, author, institution, country, and references. And then these data were imported into Microsoft Excel 2010 (Redmond, Washington, USA) to analyze the contributions of different countries, institutions, journals, and authors. VOSviewer (Leiden University, Leiden, the Netherlands) was applied to visualize the maps of coauthor-authorship, coauthor-institution, coauthor-country, co-citation references, and keywords co-occurrence. In the VOSviewer map, node size indicates the number of articles produced. The wider links between nodes means stronger cooperation strength. The color means the average publication year for the node. Blue represents the early and yellow represents the late. Citespce (Version 5.8. R1) was used to identify the keywords burst and co-cited references burst (18).

## Results

### General Data

**Figure 1:**
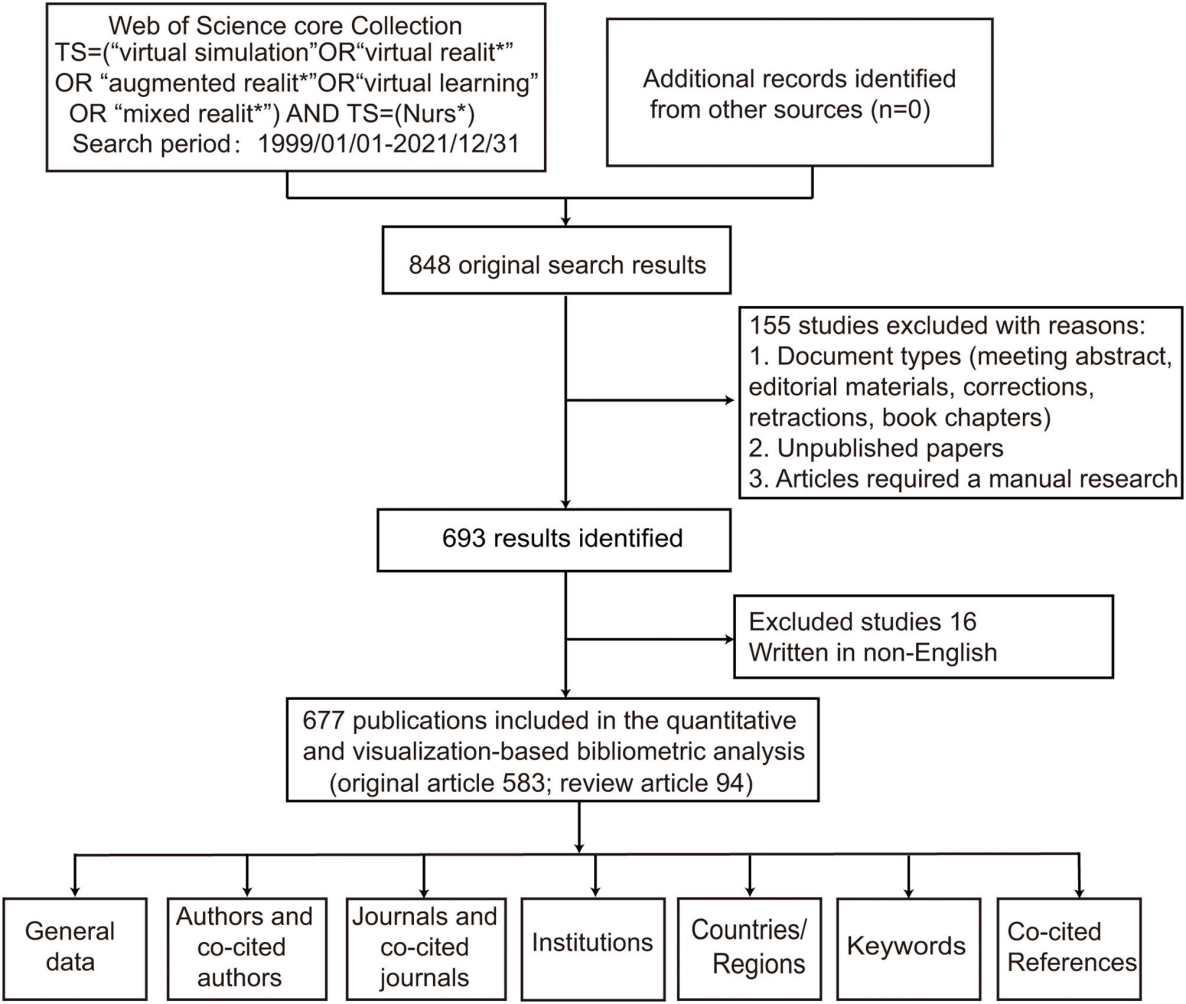
Data screening flow chart. The literature search was performed on WoSCC and language limited to English, and steps of bibliometric analysis.

**Figure 2:**
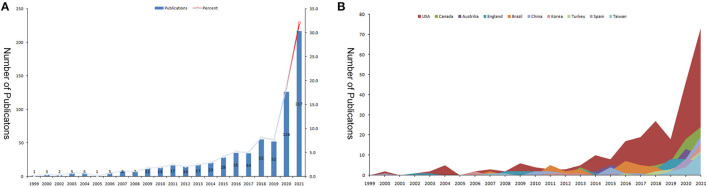
The number of publications and total citations related to virtual simulation in nursing studies. **(A)** The number of papers published and total citations each year. **(B)** The top 10 countries/regions annual publications.

An initial research of the WoSCC identified 848 publications. After excluded meeting abstract, early access and limited English language, 677 articles were finally enrolled into analysis. Original article accounts for 86.1% of the total (*n* = 583) (Figure 1). These papers were published from 1999 to 2021. The timing of publication could be divided into three periods (Phase I, 1999–2008; Phase II, 2009–2019; Phase III, 2020–2021). 1999–2008, the number of articles per year was below 10 publications. 2009–2019, the annual output of this field has grown consistently to over 50 publications. The growth is evident since 2020, with more than 100 papers published each year (126 publications in 2020 and 217 publications in 2021). In other words, more than half of papers were published in these 2 years. The peak year was 2021 (*n* = 217) (Figure 2A). The total number of citations was 8885, with 13.1 citations per paper and 47 H-index. The annual year publications of the top 10 countries were shown in Figure 2B. The USA was the major contributor in this field, and almost leading this field since its inception.

### Active Authors and Co-cited Authors

The top 10 prolific authors in this area were all from North America (Table 1). Of them, there were nine from Canada, and one from the USA. They published 84 papers and accounted for 12.4% of the total papers. Verkuyl M. from Centennial College was the most productive author in this scope with 15 publications. Followed by Luctkar-flude M from Queens University and Tyerman J from University of Ottawa with 12 papers. In terms of co-cited authors, Verkuyl, M, Hoffman HG, and Foronda, Cl were ranked the first three. Supplementary Figure 1 showed the author cooperation network and co-cited author network. There were scattered co-operations between them, and authors who work together have strong citation ties.

**Table 1:** Top 10 prolific authors and co-cited authors on virtual simulation research in nursing.

**Rank**	**Author**	**Publications**	**Citations**	**Country**	**Co-cited author**	**Co-citations**	**Country**
1	Verkuyl, M	15	167	Canada	Verkuyl, M	128	Canada
2	Luctkar-Flude, M	12	33	Canada	Hoffman, Hg	124	USA
3	Tyerman, J	12	33	Canada	Foronda, Cl	90	USA
4	Hughes, M	8	95	Canada	Inacsl Stand, Comm	78	N/A
5	Lapum, JL.	7	79	Canada	Jeffries, Pr	77	USA
6	Mastrilli, P	7	117	Canada	Cook, Da	66	USA
7	Romaniuk, D	7	120	Canada	Foronda, C	66	USA
8	St-Amant, O	6	30	USA	Padilha, Jm	51	Portugal
9	Goldsworthy, S	5	7	Canada	World Health, Organization	49	N/A
10	Mcculloch, T	5	74	Canada	Cant, Rp	47	Australia

### Active Journals and Co-cited Journals

The top 10 active and co-cited journals were identified by VOSviewer. Altogether, 269 journals contributed to VS in nursing. The top 10 most productive journals hosts 235 (34.7%) papers. Clinical Simulation in Nursing is the most productive journal (*n* = 89; 888 citations), followed by Nurse Education Today (*n* = 49; 584 citations), Journal of Nursing Education and Cin-Computers Informatics Nursing share the third position (*n* = 15). The top three co-cited journals were the same top three prolific journals, with 820, 667, 339 co-citations, respectively (Table 2).

**Table 2:** Top 10 prolific journals and co-cited journals on virtual simulation research in nursing.

**Rank**	**Journal**	**Publications**	**Citations**	IF*	**Co-cited journal**	**Co-citations**	**IF**
1	Clinical Simulation In Nursing	89	888	2.391	Clinical Simulation In Nursing	854	2.391
2	Nurse Education Today	49	584	3.442	Nurse Education Today	690	3.442
3	Cin-Computers Informatics Nursing	15	128	1.985	Journal of Nursing Education	278	1.726
4	Journal Of Nursing Education	15	116	1.726	Nursing Education Perspective	246	N/A
5	Nurse Educator	14	136	2.082	Computers & Education	225	8.538
6	Journal Of Medical Internet Research	12	219	5.428	Nurse Education In Practice	208	2.281
7	Nurse Education In Practice	12	206	2.281	Journal of Advanced Nursing	198	3.843
8	Journal Of Clinical Nursing	10	186	3.036	Journal of Medical Internet Research	197	5.428
9	Revista Latino-Americana De Enfermagem	10	74	1.886	Simulation in Healthcare-Journal of the Society for Simulation in Healthcare	194	1.929
10	Journal Of Advanced Nursing	9	97	3.843	Medical Education	176	6.251

^*^Abbreviation for impact factor (Journal Citation Reports, 2020).

### Active Institutions

**Figure 3:**
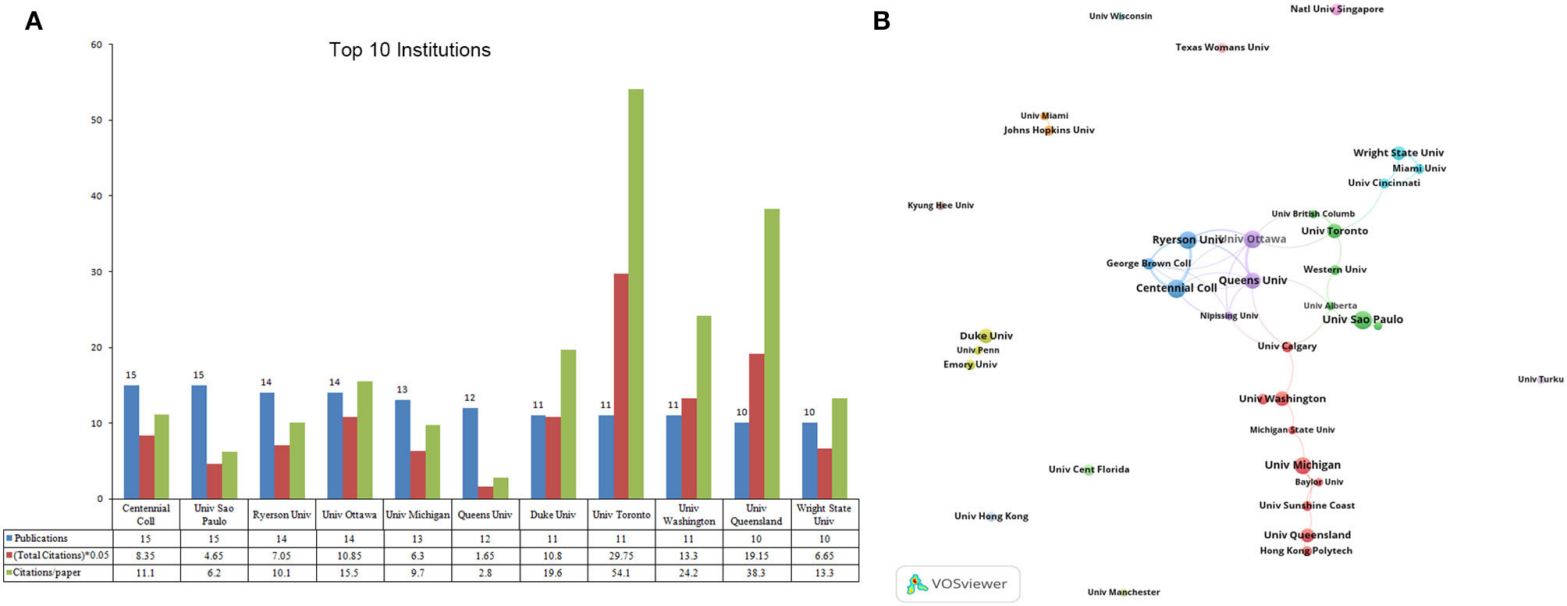
The top 10 most productive institutions and inter-institution co-operation relationships on virtual simulation in nursing research. **(A)** The number of publications, total citations, and citations per paper in top 10 institutions. **(B)** The co-authorship network visualization map of institution for virtual simulation in nursing research. Node size indicated the number of articles produced. The distance between any two nodes positively associated with the cooperation strength.

The top 10 most productive institutions were presented in Figure 3A. Among the top 10 institutions, there were five located in Canada, three in the USA, and one in Australia, Brazil, respectively. Centennial College and University of San Paulo were the most prolific institution (*n* = 15 publications), followed by University of Ottawa and Ryerson University (*n* = 14). In terms of total citations and citations per paper, University of Toronto (Canada, 595 citations, 54 citations/paper), University of Queensland (Australia, 383 citations, 38.3 citations/paper), University of Washington (Canada, 266 citations, 24.2 citations/paper) ranked in the top three. The co-authorship for organization module in VOSviewer was used to visualize the collaboration relationship among 38 institutions, which published at least five papers. As shown in Figure 3B, There were few and sporadic connecting lines between different institutions.

### Active Countries/Regions

**Figure 4:**
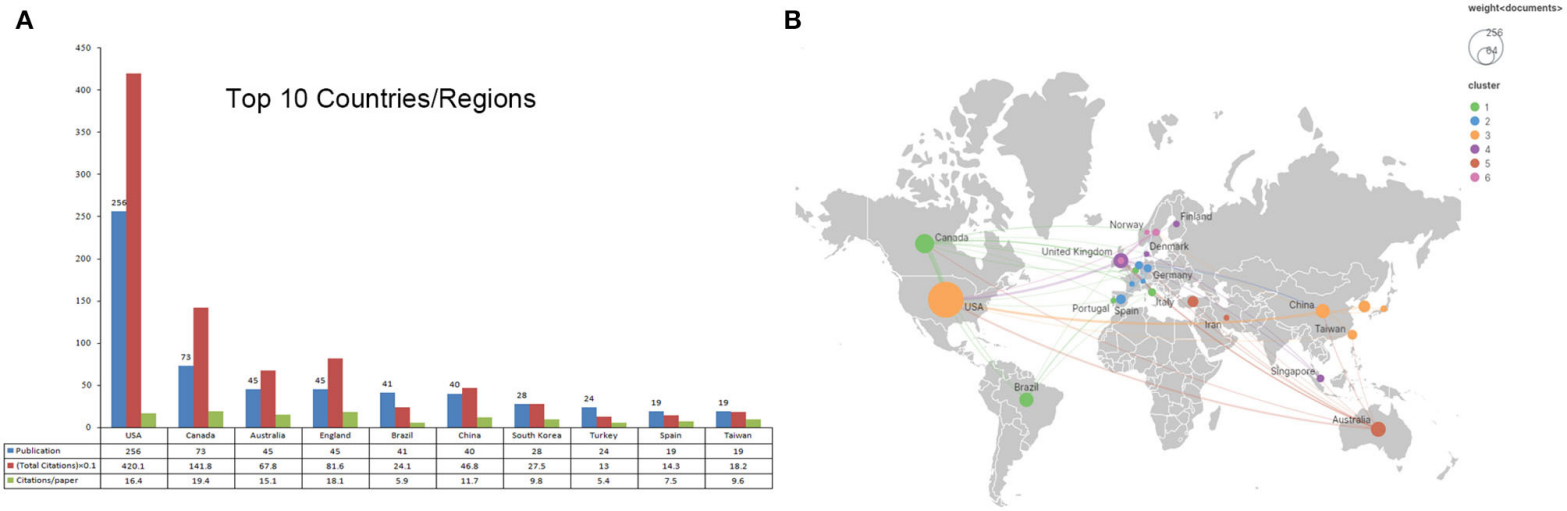
The top 10 prolific countries/regions and inter-national collaboration network on virtual simulation in nursing research. **(A)** The number of publications, total citations, and citations per paper in top 10 countries/regions. **(B)** The co-authorship network visualization map of countries for virtual simulation in nursing research. Node size indicated the number of articles produced. The distance between any two nodes positively associated with the cooperation strength. The color indicated the average publication year for the author, The blue color represented for early stage and yellow color represented late stage.

A total of 58 countries/regions participated in these publications. The top 10 were shown in Figure 4A. Obviously, the USA led in this field with 256 publications (37.8% of the total), followed by Canada (*n* = 73), Australia and England (*n* = 45). In terms of total citations, the USA ranked the first (4201), followed by Canada (1418) and England (816). In regard to citations per paper, Canada ranked first (19.4), followed by England (18.1) and the USA (16.4). The module of co-authorship for country in VOSviewer was used to map the countries' collaboration network. The smallest number of publications was settled as five, total link strength. Finally, 26 countries/regions meet our criterion. The USA, Canada, Australia, and England presented as the largest node. The strongest cooperation was between the USA and Canada and the USA and Australia (Figure 4B).

### Keywords

**Figure 5:**
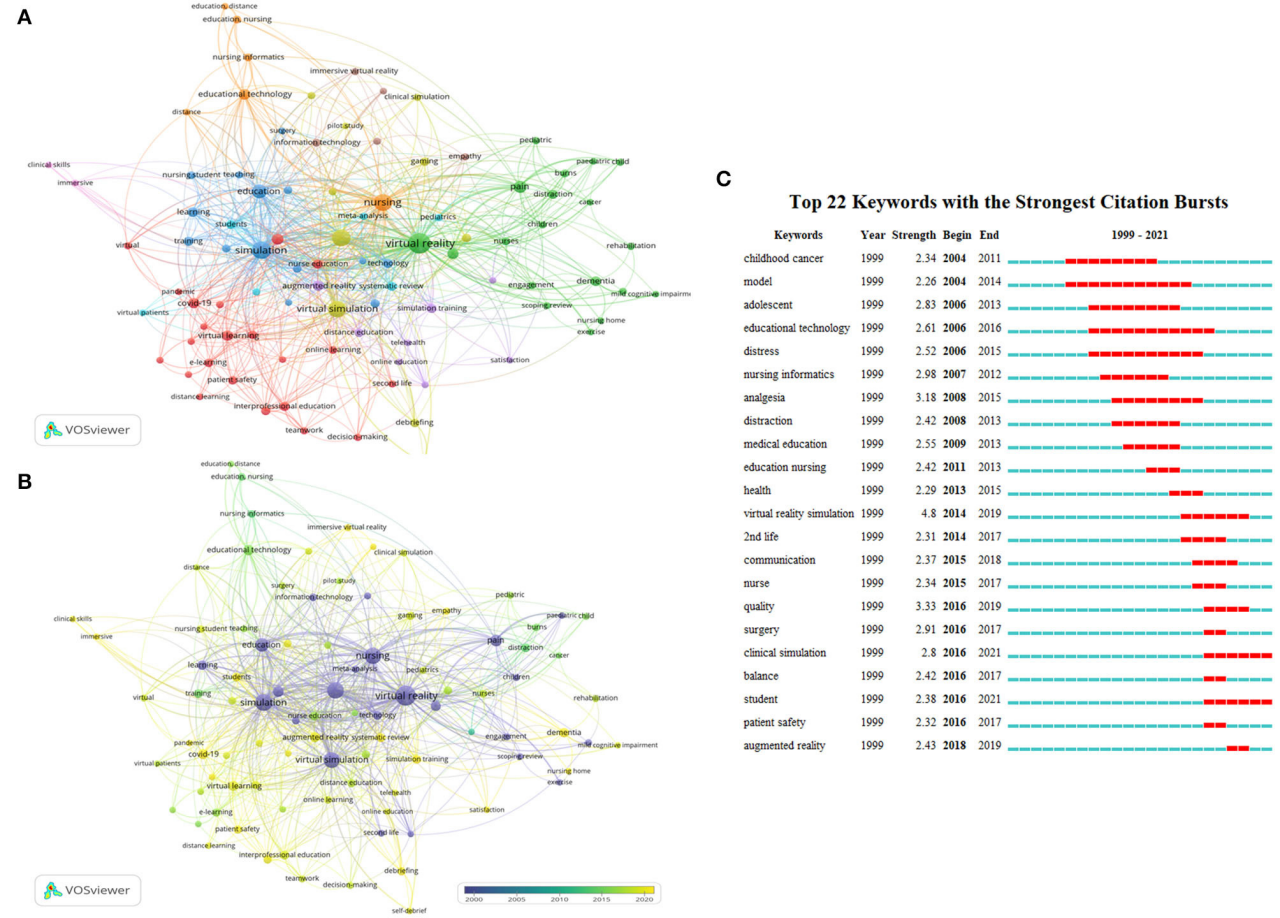
Analysis of keywords related to publications of virtual simulation in nursing field. **(A)** The co-occurrence network visualization map of keywords related to virtual simulation in nursing field. The keywords clustered into nine groups according to their color. Large nodes represented keywords with high frequency; **(B)** Keywords were colored according to the appearance for the average time. The blue color represented for early stage and yellow color represented late stage. **(C)** The top 22 keywords with the strongest citation bursts on virtual simulation in nursing field between 1999 and 2021. The red segment of the blue line denoted the burst duration of a keyword.

High-frequency keywords was usually used to describe hot-spots, and construct a knowledge map in a given field (19). We identified 1,540 keywords in total, 92 keywords occurred more than five times were enrolled into analysis. As shown in Figure 5A, the keywords were classified into nine clusters. The core keywords in the top five largest clusters, which ranked by the number of occurrences, are virtual reality (*n* = 183), simulation (*n* = 108), nurse education (*n* = 95), nursing (*n* = 92), virtual simulation (*n* = 88). As shown in Figure 5B, keywords were colored according to their average publication years. We observed although the concept of virtual simulation was built up early (colored by blue), there are lots of frontier topics spring up in recent years (colored by yellow), such as “nurse education, clinical simulation, augmented reality, and virtual learning”. Also, Citespace burst module was used to identify the research trends and shift of center topics in a given field (20). The burst duration was set to 2 years. The blue and red bar indicated infrequently and frequently cited time. The top 22 keywords with strongest citation bursts were identified and displayed in Figure 5C. Among them, virtual reality simulation has the highest burst strength (*n* = 4.8). The topics gradually shifted from “distress, cancer, model, and technology” to “education, quality, clinical simulation, and student”.

### Top Cited Articles and Co-cited References

**Figure 6:**
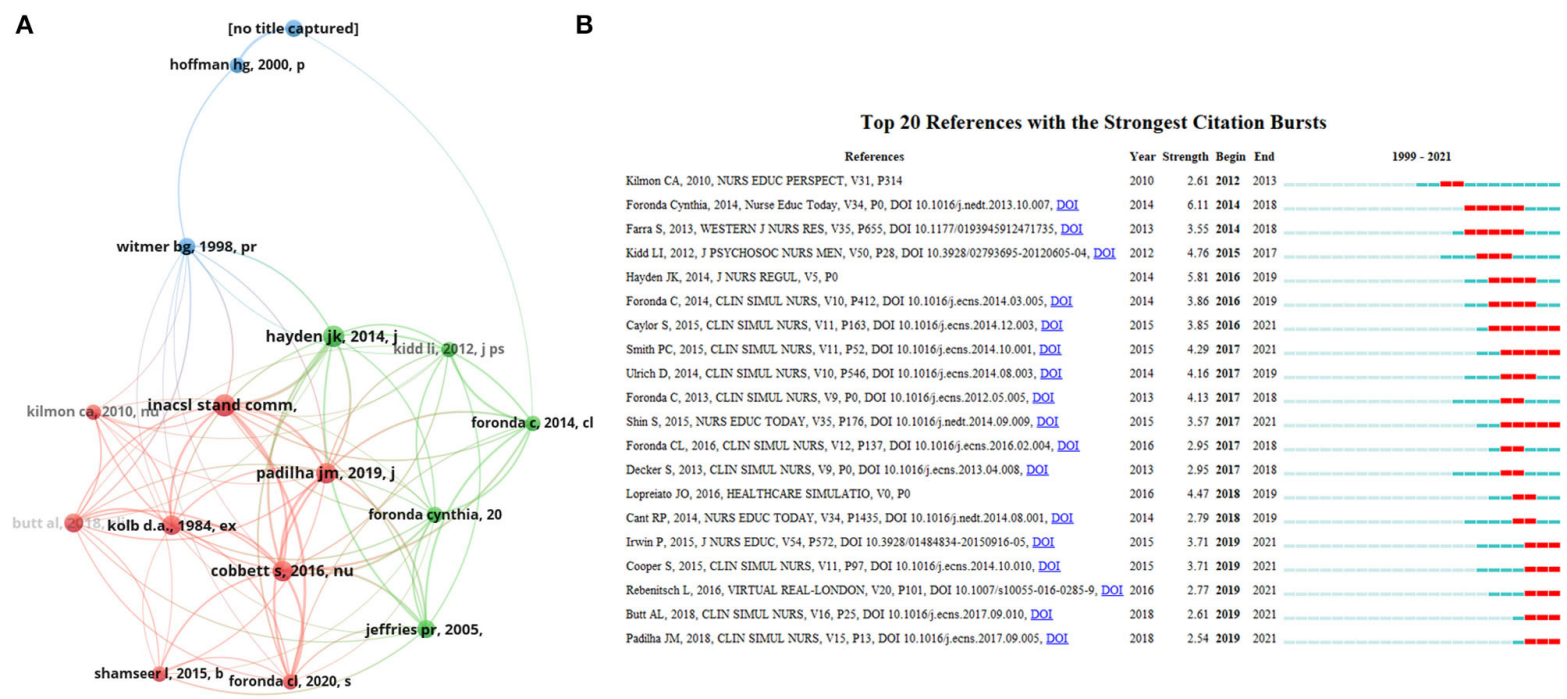
**(A)** The co-citation network visualization map of references on virtual simulation in nursing field between 1999 and 2021. **(B)** The top 20 references with the strongest citation bursts on virtual simulation in nursing field between 1999 and 2021. The blue line represented the time from its first appearance to 2021, the red line represented the burst time.

The top 10 most cited articles are listed in Table 3. The most cited article was produced by Gold et al. (21) published in *Cyberpsychology and Behavior* by 2006 with 174 citations, entitled Effectiveness of virtual reality for pediatric pain distraction during IV placement. In this study, the author reported virtual reality's positive efficacy and suitability as a pain relief tool during the pediatric intravenous placement. The co-cited reference is the article which cited by the included papers of VS in nursing, which formed the knowledge base in this field. The top16 co-cited references were identified through 20,249 references which co-cited more than 20 times by the included 677 papers. As shown in Figure 6A, the article with highest co-citations (*n* = 35) was published by INACSL Stand Comm (22) in *Clinical Simulation in Nursing* in 2016, entitled INACSL Standards of Best Practice: Simulation (SM) Simulation Design. In this work, they provide a standard framework and guideline for developing effective simulation-based experiences. Similarly, CiteSpace citation burst could identify references focused by researchers in a specific period (17, 23). The burst duration was set to 2 years. At last, 20 references with strongest citation bursts were identified in Figure 6B. Among them, Foronda Cynthia, 2014, Nurse Educ Today, V34, P0 (24) has the highest burst strength (*n* = 6.11), entitled Use of virtual clinical simulation to improve communication skills of baccalaureate nursing students: A pilot study. There are eight articles with citation bursts ending in 2021 which means they were get more attention in recent years. They are “Caylor S, 2015, CLIN SIMUL NURS, V11, P163 (25)”, “Smith PC, 2015, CLIN SIMUL NURS, V11, P52 (26)”, “Shin S, 2015, NURS EDUC TODAY, V35, P176 (27)”, “Irwin P, 2015, J NURS EDUC, V54, P572 (28)”, “Cooper S, 2015, CLIN SIMUL NURS, V11, P97 (29)”, “Rebenitsch L, 2016, VIRTUAL REAL-LONDON, V20, P101 (30)”, “Butt AL, 2018, CLIN SIMUL NURS, V16, P25 (31)”, “Padilha JM, 2018, CLIN SIMUL NURS, V15, P13 (3)”.

**Table 3:** Top 10 most cited papers related to virtual simulation research in nursing.

**Rank**	**Title**	**Journal**	**Document type**	**Corresponding author**	**Affiliation**	**Year**	**Citations**
1	Effectiveness of virtual reality for pediatric pain distraction during IV placement	Cyberpsychology & Behavior	Article	Gold, JI	Univ So Calif,	2006	174
2	Mastery Learning for Health Professionals Using Technology-Enhanced Simulation: A Systematic Review and Meta-Analysis	Academic Medicine	Review	Cook, DA.	Mayo Clin	2013	168
3	Patient Outcomes in Simulation-Based Medical Education: A Systematic Review	Journal of General Internal Medicine	Review	Cook, DA.	Mayo Clin	2013	165
4	A pilot and feasibility study of virtual reality as a distraction for children with cancer	Journal of The American Academy of Child And Adolescent Psychiatry	Article	Gershon, J	Bradley Sch S Cty	2004	166
5	Current trends in stroke rehabilitation. A review with focus on brain plasticity	Acta Neurologica Scandinavica	Review	Johansson, BB	Lund Univ	2011	170
6	Evaluation of trauma team performance using an advanced human patient simulator for resuscitation training	Journal of Trauma-Injury Infection And Critical Care	Article	Holcomb, JB	Univ Texas	2002	161
7	Virtual reality pain control during burn wound debridement in the hydrotank	Clinical Journal of Pain	Article	Hoffman, HG	Univ Washington	2008	161
8	Interprofessional communication in healthcare: An integrative review	Nurse Education in Practice	Review	Foronda, C	Johns Hopkins Univ	2016	144
9	The Insertion and Management of External Ventricular Drains: An Evidence-Based Consensus Statement	Neurocritical Care	Review	Nathan, BR	Univ Virginia	2016	149
10	Effects of distraction on pain, fear, and distress during venous port access and venipuncture in children andAdolescents with cancer	Journal of Pediatric Oncology Nursing	Article	Windich-Biermeier, A	Childrens Med Ctr	2007	140

## Discussion

The number of publications in a field reflects the activity and productivity over the years (23). We observed the overall trend of publication in this filed was upward, and could divided into three phases. Before 2008, the number of articles per year slightly increased. It may be restricted by technology such as internet access or computer popularity. Likewise, a previous study found before the year of 2005, the applications of virtual patient in medical education are small (32). Since 2020, the number of papers got a sharp increase to over 200 publications in 2021. We speculate several reasons account for this. First, because of social isolation during the COVID-19 pandemic, it is difficult for students to enter clinical practice. In addition, educators were forced to change the way of program delivery (33). Numerous online programs has emerged during the COVID-19, such as virtual lab environments (34), three-dimensional virtual world (3DVW) (35), personal protective equipment (PPE) virtual simulation games (36). Such VS programs effectively enhanced nursing student interest and provided nurse educators with novel and engaging means of content delivery (33). Second, COVID-19 pandemic accelerated the demand for more nursing staff and higher quality nursing care. Indeed, not all nurses or medical staff have the opportunity to be in the frontline battle with such an pandemic. It is difficult to educate and train these back-ups with any physical touch. However, the greatest strength of VS could provide an almost real environment that simulated any emergency in COVID-19.

Without surprising, the USA leads the VS in nursing research, which requires the collaboration of multiple disciplines, such as medical informatics, education, computer science, and software engineering. Other areas displayed the similar leading position of the USA, such as radiation-induced lung injury (37), and human-computer interaction (38), etc. Interestingly, in terms of the number of productive authors and institutions, Canada ranked ahead of the USA. Extensively studies showed that collaborations tends to be stronger between institutions or countries with shorter geographical distances (15). We speculated the cooperation links between authors and institutions within Canada were stronger than that in the USA. As expected, Supplementary Figure 1 and Figure 4B further corroborated our hypothesis. There are scattered links between numerous American authors and institutions, but relative close relationships between Canadian authors and institutions. Previous study reported studies with regional and international collaborations had a significantly higher mean number of citations than sole local collaborations. In fact, it suggested that collaborations among scholars can lead to improvements in research and contribute to greater clarity and richer insights in a field (39). Considering the scattered collaborations in this field, we hope there will be more inter-institutions and international research in the future.

Key journals, institutions, and authors provide the essential information for a given field. Clinical Simulation in Nursing, Nurse Education Today, and Journal of Nursing Education are the top three productive and co-cited journals. Researchers should continue to pay particular attention on them, because some frontier articles may be published in these journals. In addition, researchers could choose these journals for their draft submission. Institutions like University of Toronto, University of Queensland, University of Washington, and scholars like Verkuyl M, Luctkar-flude M, Tyerman J, Foronda, Cl, and Hoffman, HG should be followed and maybe the potential cooperation partner.

Keyword and reference analysis provides a graphical map of what knowledge existed and how they are interrelated in a given field. It facilitates researchers to get insight into a certain field quickly (18). The major themes generated from VOSviewer keyword co-occurrence characterize the body of knowledge structure of VS in nursing research. Specifically, the top four biggest clusters represent the whole body of this field.

### Cluster 1 (Red): Virtual Learning During COVID-19 Pandemic

The largest cluster refers to virtual learning in COVID-19 pandemic, which accounts for 23.9% of the total keywords. The primary keywords in this cluster are “nursing student (*n* = 26), virtual learning (*n* = 19), and COVID-19 (*n* = 19), nurse education (*n* = 14)” Under this cluster, researchers have explored the online virtual learning for nurse students (40–44). Studies showed their positive aspect of virtual learning. That is, virtual learning is essential at the height of the pandemic and may prove useful in other circumstances that limit clinical site availability (45, 46). Digital platforms strengthen the involvement of students (47). Herbert (48) reported their Augmented Reality (AR) app on heart failure for remote training of nursing students. Likewise, they find it could encourage students engagement during the learning process. Shamsaee et al. (49) reported virtual education had significant positive feedback on information-seeking skills and knowledge about search operators in nursing students. However, researchers also point out their worries. Amerson et al. (50) reports up to 94% of nursing students experienced a moderate level of stress in a time of virtual learning. Thus, he reminds the faculty must be attention to the mental health needs of nursing students in virtual learning during COVID-19. Also, another study discussed the negative comments regarding virtual learning from a personal level. It includes internet login and web conferencing logistics, lack of motivation to study, family difficulties, and faculty inexperience teaching in an online environment (8). Therefore, with the advent of widespread use of VS in nursing education, these problems should be fixed and pay more attention to psychological problems on students in the future.

### Cluster 2 (Green): Clinical Nursing Care Using Virtual Reality

The second largest cluster refers to cure or clinical use for virtual reality. It includes 20.6% keywords of the total. The primary keywords in this cluster are “virtual reality (*n* = 183), pain (*n* = 33), anxiety (*n* = 20), dementia (*n* = 18), distraction (*n* = 15).” Under this cluster, researchers focused on its clinical applications. Studies from Nilsson et al. (51) and Gershon et al. (52) showed that VR could effectively relieve the needle-related procedural pain and distress in children and adolescents with cancer diseases. Also, studies reported VR could effectively reduce children's pain and distress during flu vaccinations (53) and burn wound care procedures (54). Apart from the applications for caring for children, older people also benefit from VR programs. Loggia et al. (55) reported the use of VR could help able-bodied older people to achieve physical activity recommendations, even with moderate cognitive impairments. Davis and Ohman (56) and Hannans et al. (57) reported VR could help persons with Alzheimer's disease find their way more effectively, help them maintain independence and enhance their cognitive and affective knowledge. Brimelow et al. (58) reported VR could reduce apathy and improve mood in aged care. Although many benefits may get from VR in older people's nursing, difficulties still exist in dementia older people caring, like less empathy in nurses. Previous studies show VR could enhance nursing students' effectiveness and interest in working with older people (59). Also, Campbell et al. (60) reported using a VR dementia experience system could increase nursing student awareness, knowledge, and sensitivity of Alzheimer's disease. Further exploration is how these VR programs translate into improved care for those older people.

### Cluster 3 (Blue): Education in Nurse Practitioners Using VS

The third-largest cluster related to education in nurse practitioners using VS. It includes 15.2% keywords of the total. The primary keywords in this cluster are ”simulation (*n* = 108), education (*n* = 51), learning (*n* = 16), training (*n* = 15).“ Under this cluster, researchers focused on clinical education in nurse practitioners. Tsai et al. (61) developed a computer-assisted protocol using VR in performing Port-A catheter inserting. Results suggested it could reduce fear of performing the Port-A catheter technique, significantly reduce error rates and increase correct equipment selection in novice nurses. Samosornet al. (62) developed a VR airway Laboratory to teach difficult airway management skills to nursing students. For cardiopulmonary resuscitation (CPR), researchers explored the effects of VR (63) and AR (64) in training nursing students. Compared with the traditional teaching approach, VR and AR CPR training systems get positive feedback as experienced in a realistic environment. Again, studies report the 3D virtual environment such as 2nd Life laboratory is a good way to practice the students' experience of learning decision-making skills (65–68). Another virtual simulation program is virtual patients. It developed to provide a realistic standard clinical situation to train the ability of nursing students [e.g., clinical reasoning (69–71), communication skills (72–74) situation awareness, and teamwork capability (75)]. However, there are still lingering questions remain to elucidate. Currently, the predominant methods for assessing learning outcomes are combinations of paper-based exams and observations from clinical teachers (76), effective and objective assessment methods related to learning outcomes are still lacking (77–79). Thus, as a promising teaching approach, we call for nurse managers, policymakers, and nurse educators to develop more VS programs to train nursing students and build more reliable and objective assessment methods to validate learning outcomes.

### Cluster 4 (Orange): Cluster 4 (Orange): Education Technology in VS

The fourth cluster associated with the education technology for nursing research, which consists of 6.3% keywords of the total. The primary keywords are “nursing (*n* = 92) and educational technology (*n* = 24)”. Under this cluster, researchers focused on technologies on VS. The major virtual technologies used in nursing education are VR and AR. VR technologies have three common features: “(1) immersion, (2) perception to be present in an environment, (3) interaction with that environment.” AR technologies have three defining characteristics: “(1) combines real and virtual, (2) interactive in real-time, (3) registered in 3D.” The main difference between VR and AR is that AR merged the real world with virtual experience (80). Different terms [e.g., virtual learning spaces (81); virtual worlds (82); immersive three-dimensional (3D) interactive video program (6)] have been used to refer to VR and AR technologies, and sometimes the distinction between VR and AR is unclear. Thus, there is a call to unify the definition of VR and AR (1). Contrary to being used with VR and AR in education, MR is less used, but recently got concerned. Wunder et al. (83) using an AR headsets to simulate fire in the operating room to train nurse anesthesia students. Although VR, AR, and MR provide a chance to revolutionize nursing education delivery, and promote student-centered learning (84). There are some shared challenges among the three virtual technologies. (1) Faculty and their institutions involved need to be able to invest significant time, money, and resources to successfully develop and launch VS technologies for nursing education. (2) Faculty need to be adequately trained before using VS technology to manage and prevent VR technology difficulties (e.g., poor video and sound quality, poor network connectivity, low fidelity of the virtual experience, or computer problems). (3) End-users for virtual technologies may experience cybersickness or digital motion sickness (e.g., feeling disoriented, dizzy, nauseous, and sore eyes). These issues warrant further research in the future.

## Limitations

First, the papers on virtual simulation in nursing were searched based on the WoSCC. Although WoSCC is recognized as one of the most authoritative databases, PubMed, Scopus, and Google scholar are also widely accepted by scientists. Second, the number of citations and citations per paper are influenced by time and remain controversial as a comprehensive indicator of the quality of one paper or the author. Likewise, the larger number of publications was not the only indicator of influence for the journal, as other indicators (e.g., impact factor, SNIP, CiteScore, SJR) are widely used (85). Third, we included only English papers in this study, several papers with non-English languages were excluded, such as Chinese, Portuguese (*n* = 8), and Spanish (*n* = 4). Finally, database updates may result in discrepancies.

## Conclusions

This bibliometric analysis identified major contributing authors, institutions, countries, journals and mapped the knowledge network of virtual simulation in nursing research. The research hotspot is gradually shifting from clinical nursing care to studies of nursing education using different virtual simulation technologies. Further research directions are as following: (1) Strengthen the co-operation between authors, institutions, and countries. (2) Reducing psychological problems and physical sickness of end-users. (3) Developing more VS programs to train nursing students and build more reliable and objective assessment methods to validate learning outcomes. (4) Strengthen the training of faculty abilities to VS technology to manage and prevent VS technology difficulties.

## Data Availability Statement

The original contributions presented in the study are included in the article/Supplementary Material, further inquiries can be directed to the corresponding author/s.

## Author Contributions

QZ conceived of the study, participated in its design, and drafted the manuscript. JL involved in study design, obtained data and contributed to interpretation, and helped to draft the manuscript. JC provided the theoretical frameworks and performed much of the editing of the manuscript. All authors read and approved the final manuscript.

## Funding

This study was supported by the Hunan Science and Technology Innovation Platform and Talent Plan (Grant: 2017TP1004).

## Conflict of Interest

The authors declare that the research was conducted in the absence of any commercial or financial relationships that could be construed as a potential conflict of interest.

## Publisher's Note

All claims expressed in this article are solely those of the authors and do not necessarily represent those of their affiliated organizations, or those of the publisher, the editors and the reviewers. Any product that may be evaluated in this article, or claim that may be made by its manufacturer, is not guaranteed or endorsed by the publisher.

## Supplementary Material

Supplementary Figure 1 Author co-operation network and co-cited author network on virtual simulation in nursing research. **(A)** Co-authorship network visualization map of authors for virtual simulation in nursing field. **(B)** Co-cited author network visualization map of virtual simulation in nursing field. Node size indicated the number of articles produced. The distance between any two nodes positively associated with the cooperation strength.

## Abbreviations

WoSCC: Web of Science core collection
VS: virtual simulation
VR: virtual reality
AR: augmented reality
MR: mixed reality
IF: impact factor
CPR: cardiopulmonary resuscitation.
