# Supplementary figures and images for: Global Trends and Hot-Spots in Research on Virtual Simulation in Nursing: A Bibliometric Analysis From 1999 to 2021
Source: Front Public Health. 2022 Apr 25;10:890773. doi: 10.3389/fpubh.2022.890773 (PMC9082269; doi:10.3389/fpubh.2022.890773)

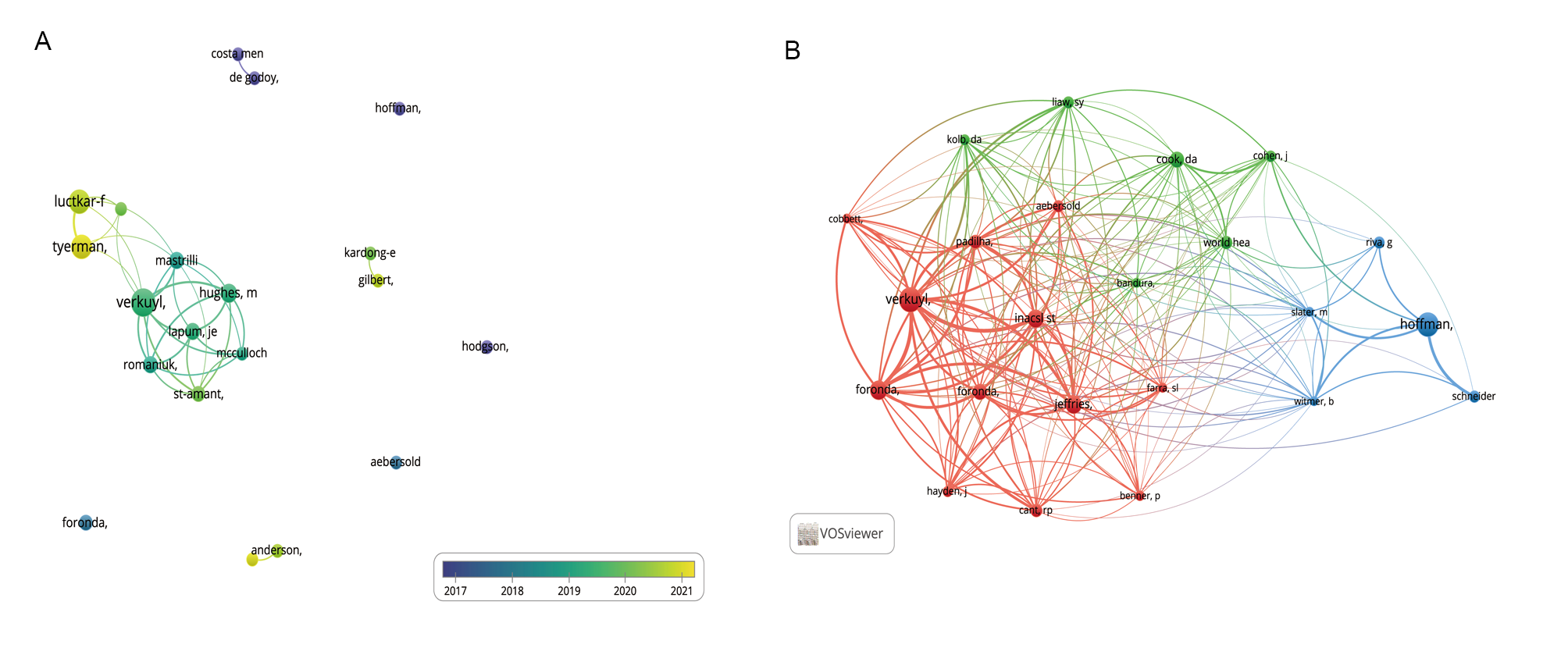

Supplement: Supplementary Figure 1 — Author co-operation network and co-cited author network on virtual simulation in nursing research. (A) Co-authorship network visualization map of authors for virtual simulation in nursing field. (B) Co-cited author network visualization map of virtual simulation in nursing field. Node size indicated the number of articles produced. The distance between any two nodes positively associated with the cooperation strength. [file Image_1.TIF]
